# Development and validation of a predictive model for PACU hypotension in elderly patients undergoing sedated gastrointestinal endoscopy

**DOI:** 10.1007/s40520-024-02807-6

**Published:** 2024-07-18

**Authors:** Zi Wang, Juan Ma, Xin Liu, Ju Gao

**Affiliations:** 1 Department of Anesthesiology, Northern Jiangsu People’s Hospital Affiliated to Yangzhou University, Jiangsu Yangzhou, 225001 China; 2 Yangzhou University, Jiangsu Yangzhou, 225001 China

**Keywords:** Aged, Endoscopy, Gastrointestinal, Recovery room, Risk factors

## Abstract

**Background:**

Hypotension, characterized by abnormally low blood pressure, is a frequently observed adverse event in sedated gastrointestinal endoscopy procedures. Although the examination time is typically short, hypotension during and after gastroscopy procedures is frequently overlooked or remains undetected. This study aimed to construct a risk nomogram for post-anesthesia care unit (PACU) hypotension in elderly patients undergoing sedated gastrointestinal endoscopy.

**Methods:**

This study involved 2919 elderly patients who underwent sedated gastrointestinal endoscopy. A preoperative questionnaire was used to collect data on patient characteristics; intraoperative medication use and adverse events were also recorded. The primary objective of the study was to evaluate the risk of PACU hypotension in these patients. To achieve this, the least absolute shrinkage and selection operator (LASSO) regression analysis method was used to optimize variable selection, involving cyclic coordinate descent with tenfold cross-validation. Subsequently, multivariable logistic regression analysis was applied to build a predictive model using the selected predictors from the LASSO regression. A nomogram was visually developed based on these variables. To validate the model, a calibration plot, receiver operating characteristic (ROC) curve, and decision curve analysis (DCA) were used. Additionally, external validation was conducted to further assess the model’s performance.

**Results:**

The LASSO regression analysis identified predictors associated with an increased risk of adverse events during surgery: age, duration of preoperative water abstinence, intraoperative mean arterial pressure (MAP) <65 mmHg, decreased systolic blood pressure (SBP), and use of norepinephrine (NE). The constructed model based on these predictors demonstrated moderate predictive ability, with an area under the ROC curve of 0.710 in the training set and 0.778 in the validation set. The DCA indicated that the nomogram had clinical applicability when the risk threshold ranged between 20 and 82%, which was subsequently confirmed in the external validation with a range of 18–92%.

**Conclusion:**

Incorporating factors such as age, duration of preoperative water abstinence, intraoperative MAP <65 mmHg, decreased SBP, and use of NE in the risk nomogram increased its usefulness for predicting PACU hypotension risk in elderly patient undergoing sedated gastrointestinal endoscopy.

**Supplementary Information:**

The online version contains supplementary material available at 10.1007/s40520-024-02807-6.

## Introduction

Gastrointestinal endoscopy is becoming increasingly common in many countries as a means of early cancer screening in adult patients [1, 2]. To enhance patient comfort and improve the accuracy of the examination, sedative drugs are commonly administered. In China, propofol is the most frequently used sedative [3] owing to its rapid onset, strong sedative effect, short half-life, rapid recovery, and high patient satisfaction [4]. However, several studies have reported that complications such as hypotension and hypoxemia frequently occur with propofol sedation [5–7], especially in elderly patients [8–10].

The dosage of propofol administered during endoscopy is typically greater than the minimum sedation required for the procedure [11–14]. Studies indicate that deep sedation with propofol is more likely to result in hypotension compared to light sedation during endoscopy [15, 16]. A study involving 2132 patients undergoing endoscopy in Melbourne found that 23% of patients who received propofol sedation experienced a major unplanned event, with 11.8% of these events caused by significant hypotension (defined as systolic blood pressure <90 mmHg), occurring in 8% of patients [9]. Therefore, propofol-induced hypotension is the most common sedation-related complication [17]. Although the hypotension is usually mild and short-lived, recent research has shown that even brief episodes of intraoperative and postoperative hypotension are associated with adverse postoperative outcomes such as increased mortality, myocardial injury, acute kidney injury, and postoperative stroke in patients receiving general anesthesia for noncardiac surgery [18, 19]. This can lead to prolonged hospital stays and decreased patient survival rates [20]. Notably, intraoperative and postoperative hypotension during gastrointestinal endoscopy are often overlooked or undetected, despite the short duration of the procedure. Therefore, identifying high-risk factors for hypotension prior to endoscopy is clinically significant and can help physicians intervene proactively, stratify the risk of hypotension, and ensure patient safety and comfort.

To date, research efforts aimed at developing and validating predictive models for perioperative hypotension have largely focused on the intraoperative period [21–25]. While the link between intraoperative hypotension and adverse outcomes is well-established, recent studies have highlighted the significant burden of organ dysfunction and mortality resulting from hypotensive events occurring early in the postoperative period [26–29]. In addition, a follow-up investigation demonstrated that hypotensive events during this time can be exceptionally prolonged and severe, yet they often go unrecognized and untreated [29]. Therefore, the ability to predict hypotensive events in the post-anesthesia care unit (PACU) has the potential to improve both patient and population-level outcomes by facilitating interventions that reduce the severity or duration of postoperative hypotension [21, 22].

The available literature reveals a lack of information regarding the identification of clinical predictors that could potentially cause hypotension in patients undergoing sedated gastrointestinal endoscopy within the PACU. Consequently, the current study aims to investigate and evaluate clinical predictors of PACU hypotension in such patients, with a goal to develop a comprehensive multivariate model that accurately predicts the incidence of PACU hypotension in patients undergoing sedated gastroenterology procedures.

## Materials and methods

### Inclusion and exclusion criteria

A prospective observational study was conducted at the Endoscopy Center in Northern Jiangsu People’s Hospital, which is affiliated with Yangzhou University, from March to December 2021. Eligible participants included individuals of any gender, aged 65 years or older, with an American Society of Anesthesiologists (ASA) classification of III or lower, a body mass index (BMI) below 30 kg/m^2^, those with complete cognitive function, and those who provided informed consent. Exclusion criteria were therapeutic endoscopy, inadequate bowel preparation, use of other anesthetic drugs such as diazoxide or fentanyl during the procedure, pre-existing organ failure at the time of data collection, an ASA classification of 4 or higher, and incomplete data.

### Sample size

The sample size was calculated for the primary study. As this is a retrospective analysis focusing on prospectively gathered anonymized data, a specific sample size was not calculated.

### Patient monitoring and sedative intervention

All included patients underwent routine gastrointestinal preparation before surgery. This included a fasting period of 6–8 h and abstaining from water for 4 h before the procedure. A pre-anesthesia assessment was conducted, and informed consent was obtained from each patient at the anesthesia clinic. Those undergoing colonoscopy also received a compound polyethylene glycol electrolyte solution for bowel clearance. After admission, intravenous access was established, and vital signs, including heart rate (HR), systolic blood pressure (SBP), diastolic blood pressure (DBP), and oxygen saturation (SpO_2_), were routinely monitored at 3-min intervals. In addition, open-mask oxygen was administered at a rate of 3–5 L/min. Gastroenterologists and anesthesiologists who were qualified in accordance with appropriate diagnosis and treatment practices performed the gastrointestinal operations and administered anesthesia. Anesthesia induction was achieved with slow intravenous administration of propofol at a dose of 1.5 mg/kg, along with remifentanil at a dose of 0.5 μg/kg. Maintenance of anesthesia was managed with slow intravenous administration of propofol at a dose of 0.5 mg/kg every 5 min. If the patient exhibited intraoperative body movement or signs of shallow anesthesia, additional propofol at a dose of 0.5 mg/kg and remifentanil at a dose of 0.3 μg/kg were administered slowly until stable anesthesia was achieved. If the patient’s intraoperative SBP was less than 90 mmHg or 20% of the baseline value, NE at a dose of 8–16 μg was slowly administered intravenously. If the patient’s HR was less than 45 beats/min, atropine at a dose of 0.5 mg was slowly administered intravenously. In cases of hypoxemia (SpO_2_ ≤ 90%), appropriate pain stimulation, such as holding the jaw or lightly pressing the mandibular angle, was applied, and if necessary, the operation was paused, and oxygen was administered using a pressure mask. At the end of the procedure, patients were transferred to the PACU for awakening. The resuscitation level was assessed every 5 min using the postanesthetic discharge scoring system (PDASS), which is the standard discharge score at the endoscopy center. A PDASS score greater than 9 was considered to indicate sufficient anesthetic recovery and was used as a criterion for discharge from the PACU.

### Data collection and definitions

Based on a thorough review of relevant literature, an extensive compilation of prior studies, and comprehensive clinical observations, a set of factors that may potentially affect the duration of PACU retention time was identified. These factors were integrated into a survey scale. Upon patients’ arrival in the waiting area, the investigators obtained information on their general health condition and medical history through direct, face-to-face questioning. During the anesthesia process, anesthesiologists documented the intraoperative conditions, whereas the medical staff in the PACU monitored and recorded the patients’ postoperative recovery status.

The study recorded several patient characteristics, including age, gender, BMI, ASA classification, frailty status, history of hypertension, history of diabetes mellitus, alcohol consumption, smoking history, and habitual snoring status. These factors were documented before the endoscopy procedure. During and after the procedure, data on vital signs, medication administration, and any adverse events were also collected. For this study, participants were classified as alcohol consumers if they had consumed alcoholic beverages at least once per week during the preceding six months [30]. Similarly, participants were classified as cigarette smokers if they had smoked at least 10 cigarettes per week during the preceding six months. Habitual snoring was defined as snoring for more than three nights per week. The degree of frailty was assessed using the Fried scale, with scores of ≥3 indicating frailty, scores of 1–2 indicating pre-frailty, and scores of 0 indicating normal health. The study also evaluated patients’ emotional states using the Hamilton Anxiety Inventory and Hamilton Depression Inventory, with scores of >1 indicating positive emotions and scores of ≤1 indicating negative emotions.

PACU hypotension is typically characterized by an SBP of less than 90 mmHg for any duration or a decrease of at least 20% from the preoperative baseline in at least one measurement taken in the PACU. These definitions are consistent with the established clinical escalation workflows in healthcare facilities, as even minor instances of hypotension can result in adverse outcomes.

### Compliance with ethical guidelines

The study was approved by the Ethics Committee of Northern Jiangsu People’s Hospital, affiliated with Yangzhou University (approval number: 2021ky024) and registered with the China Clinical Trial Registration Center (Registration Number: ChiCTR210046351). Prior to participation, all patients provided written informed consent. The study adhered to the ethical principles outlined in the Declaration of Helsinki.

### Statistical analyses

Statistical analysis was conducted using R software (version 4.2.1; R). Patient characteristics were compared between groups with and without PACU hypotension. To compare proportions, the chi-square test was used. For continuous variables, the independent samples *t*-test or Mann–Whitney *U* test was used for normally and non-normally distributed data, respectively. A *P *value <0.05 was considered to indicate statistical significance.

Initially, a total of 2919 patients who underwent sedated gastrointestinal endoscopy were enrolled in the study. The R *caret* package was used to randomly divide them into a training set consisting of 2190 participants and a validation set comprising 729 participants, maintaining a proposed ratio of 3:1. The least absolute shrinkage and selection operator (LASSO) regression analysis was conducted using the R ***glmnet*** package, which is a linear regression model utilized for variable selection and shrinkage. The objective of the LASSO regression analysis was to identify a subset of predictors that could minimize prediction error for the quantitative response variables. This was achieved by imposing constraints on the model parameters, causing the regression coefficients for specific variables to shrink toward zero. Variables with a regression coefficient equal to zero after the shrinkage process are excluded from the model while variables with non-zero regression coefficients variables are most strongly associated with the response variable. The family parameter was set to “binomial” to account for the binary nature of the dependent variable in the analysis, namely the presence of PACU hypotension. Subsequently, the Bias type measure (−2 loglikelihood) was employed in the LASSO regression analysis, based on the binomial family and −2 loglikelihood type measure. The analysis was conducted using ten-fold cross-validation in the R software, with centralization and normalization of the variables included in the model, and the best lambda value was selected. The model that showed good performance with a minimal number of independent variables was denoted as *Lambda.Lse*. The training set was used to identify the optimal predictors for current risk factors using the LASSO method. These included gender, age, frailty status, hypertension, diabetes, BMI, smoking, alcohol consumption, changes in heart rate and blood pressure during surgery, and the amounts of sedative and analgesic medication administered. The initial screening of variables was conducted using the aforementioned inclusion criteria.

Logistic regression was conducted using the R *rms* package to construct a predictive model for hypotension risk in the PACU. The multivariate logistic regression analysis employed the characteristics identified in the initial phase of the LASSO regression model, which were assessed as odds ratios (ORs) with 95% confidence intervals (CIs) and p-values. All statistical significance levels were set as two-sided. After importing the selected characteristics and analyzing their statistical significance levels, we used the predictors of significance to develop a predictive model for PACU hypotension risk. In our study, all selected features were found to be statistically significant and were thus incorporated into the development of the nomogram prediction models. A nomogram prediction model was also developed using the R *rms* package with all statistically significant variables.

Several validation methods were used to estimate the accuracy of the risk prediction model, including the ROC curve analysis performed using the *pROC* package in R. The area under the ROC curve (AUC) was used to evaluate the discrimination between true positives and false positives, thereby assessing the quality of the risk nomogram. Calibration curves for the PACU hypotension risk nomogram were plotted and calculated using the rms package, accompanied by Hosmer–Lemeshow tests (HLtest.R) to evaluate the calibration. In addition, decision curve analysis (DCA) was performed using the R *nricens* package to determine the clinical utility of the nomogram based on the net benefit at different threshold probabilities in the Geriatric sedated Gastrointestinal Cohort.

## Results

**Fig. 1:**
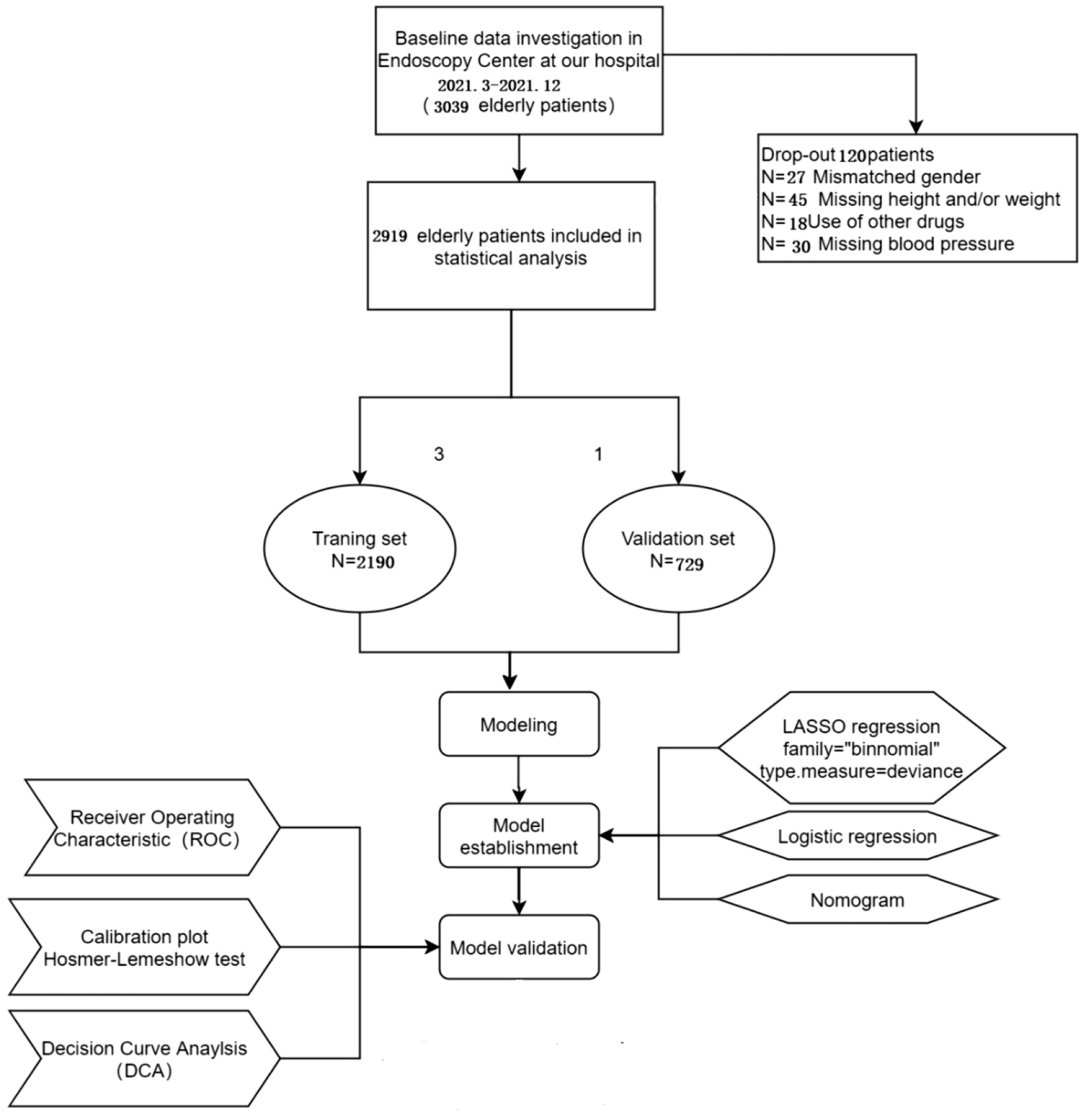
Flow diagram of study design

During this period, we collected the data of 3039 elderly patients undergoing sedated gastrointestinal endoscopy. However, 120 patients were excluded due to loss to follow-up, missing or incorrect data, and/or missing samples, resulting in a loss rate of 3.9%. Thus, the final analysis included 2919 elderly patients. The flowchart of the study design is presented in Fig. 1.Of the 2919 patients included in the study, 1533 were men and 1386 were women, with a median age of 68 years (range, 65–73 years). Gastroscopy was performed in 1389 (47.6%) patients, colonoscopy in 801 (27.4%) patients, and both procedures in 729 (24.9%) patients. PACU hypotension occurred in 1134 (38.8%) patients, and no cases of tracheal intubation were observed in the entire cohort. To assess the predictive accuracy of the risk nomogram, we randomly divided the 2919 samples into training and validation sets at a ratio of 3:1. The training set comprised 2190 patients, and the validation set included 729 patients. Table 1 presents the primary characteristics of the patients in both sets.

**Table 1:** Characteristics of patients undergoing sedated gastroenteroscopy according to presence/absence of PACU hypotension and randomization

Items	Total patient cohort (n = 2919)	PACU hypotension (n = 1134)	PACU no hypotension (n = 1785)	Training set (n = 2190)	Validation set (n = 729)	*P* value
Age, (years)	69 [65–73]	69 [65–74]	68 [65–73]	68 [65–73]	69 [65–73]	0.931
Male sex, n (%)	1533 (52.5)	577 (50.8)	976 (53.6)	1152 (52.6)	381 (52.3)	0.986
BMI, (kg/m^2^)	23.42 ± 3.22	23.33 ± 3.26	23.48 ± 3.19	23.39 ± 3.22	23.54 ± 3.22	0.604
ASA class, n (%)						
I	612 (21.0)	258 (22.8)	354 (19.8)	438 (20.0)	174 (23.9)	0.438
II	1887 (64.6)	678 (59.8)	1209 (67.7)	1434 (65.5)	453 (62.1)	
III	420 (14.4)	198 (17.5)	222 (12.4)	318 (14.5)	102 (14.0)	
Frailty status, n (%)						0.091
Pre-frailty	1557 (53.3)	606 (53.4)	957 (53.3)	1209 (55.2)	348 (47.7)	
Frailty	513 (17.5)	201 (17.7)	312 (17.5)	381 (17.4)	132 (18.1)	
Vertigo, n (%)	771 (26.4)	282 (24.9)	489 (27.4)	603 (27.5)	168 (23)	0.197
Motion sickness, n (%)	408 (13.9)	174 (15.3)	234 (13.1)	318 (14.5)	90 (12.3)	0.459
Fatigue, n (%)	711 (24.3)	288 (25.4)	423 (23.7)	558 (25.5)	153 (21)	0.185
Allergy, n (%)	112 (11.5)	50 (13.2)	62 (10.4)	81 (11.1)	31 (12.8)	0.557
Hypertension, n (%)	1221 (41.8)	429 (37.8)	792 (44.4)	909 (41.5)	312 (42.8)	0.781
Diabetes mellitus, n (%)	429 (14.6)	180 (15.9)	249 (13.9)	330 (15.1)	99 (13.6)	0.643
Liver disease, n (%)	288 (9.8)	102 (9.0)	186 (10.4)	213 (9.7)	75 (10.3)	0.565
Kidney disease, n (%)	9 (0.3)	3 (0.3)	6 (0.3)	8 (0.4)	1 (0.1)	0.577
Exercise habits, n (%)	1950 (66.8)	771 (68.0)	1179 (66.1)	1488 (67.9)	462 (63.4)	0.218
Alcohol consumption, n (%)	615 (21.0)	198 (17.5)	417 (23.4)	477 (21.8)	138 (18.9)	0.394
Smoking, n (%)	630 (21.5)	231 (20.4)	399 (22.4)	365 (21.2)	165 (22.6)	0.712
Habitual snoring, n (%)						0.998
Mild	1416 (48.5)	564 (49.7)	852 (47.7)	312 (48.5)	354 (48.6)	
Moderate	303 (10.3)	90 (7.9)	213 (11.9)	225 (10.3)	78 (10.7)	
Severe	105 (3.5)	39 (3.4)	66 (3.7)	81 (3.3)	24 (3.3)	
Fasting time (h)	17.52 [15.51–21.22]	16.89 [15.21–20.92]	17.77 [15.65–21.77]	17.63 [15.41–21.14]	17.02 [15.61–21.33]	0.964
Water abstinence time (h)	15.9 [7.6–16.5]	14.68 [12.45–16.25]	12.70 [6.93–16.77]	14.04 [7.73–16.71]	13.5 [7.42–16.11]	0.062
Endoscopy, n (%)						0.165
Gastroscopy only	1389 (47.6)	609 (53.7)	840 (43.7)	1008 (46)	381 (52.3)	
Colonoscopy only	801 (27.4)	279 (24.6)	522 (29.2)	606 (27.7)	195 (26.7)	
Gastroscopy and colonoscopy	729 (24.9)	246 (21.7)	483 (27.1)	576 (26.3)	153 (21.0)	
Total propofol dose (mg/kg)	2.08 ± 0.69	2.03 ± 0.64	2.12 ± 0.72	2.07 ± 0.71	2.11 ± 0.67	0.221
Total propofol dose (mg/[kg*min])	0.27 ± 0.21	0.29 ± 0.20	0.26 ± 0.22	0.28 ± 0.22	0.27 ± 0.21	0.897
Total remifentanil dose (μg/kg)	0.30 [0.25–0.36]	0.30 [0.25–036]	0.30 [0.25–0.34]	0.30 [0.25–0.36]	0.30 [0.25–0.35]	0.351
Total remifentanil dose (μg/[kg*min])	0.03 [0.02–0.06]	0.04 [0.02–0.06]	0.03 [0.02–0.05]	0.03 [0.02–0.06]	0.04 [0.02–0.06]	0.447
Use of NE, n (%)	1290 (44.1)	339 (29.9)	951 (53.3)	936 (42.7)	354 (48.6)	0.132
SBP change (%)	0.27 ± 0.14	0.28 ± 0.14	0.26 ± 0.14	0.28 ± 0.13	0.28 ± 0.13	0.951
DBP change (%)	0.31 ± 0.16	0.31 ± 0.16	0.30 ± 0.16	0.30 ± 0.16	0.31 ± 0.15	0.688
HR change (%)	0.07 ± 0.14	0.06 ± 0.15	0.07 ± 0.14	0.13 ± 0.09	0.12 ± 0.1	0.506
Intraoperative MAP <65 mmHg, n (%)	1296 (44.4)	588 (51.9)	708 (39.7)	957 (43.7)	339 (46.5)	0.571
Intraoperative adverse events, n (%)						
Hypotension	1791 (61.4)	762 (67.2)	1029 (57.6)	1217 (60.1)	474 (65)	0.201
Hypoxemia	435 (14.9)	180 (15.9)	255 (14.3)	300 (13.7)	135 (18.5)	0.085
Body movement	105 (3.5)	33 (2.9)	72 (4.0)	87 (4.0)	18 (2.5)	0.373
Coughing	39 (1.3)	15 (1.3)	24 (1.3)	30 (1.4)	9 (1.2)	0.999

*P *value <0.05: Comparison between training set and validation set; *BMI* body mass index, *SBP/DBP* systolic/diastolic blood pressure, *NE* norepinephrine, *MAP* mean arterial pressure, *HR* heart rate

**Fig. 2:**
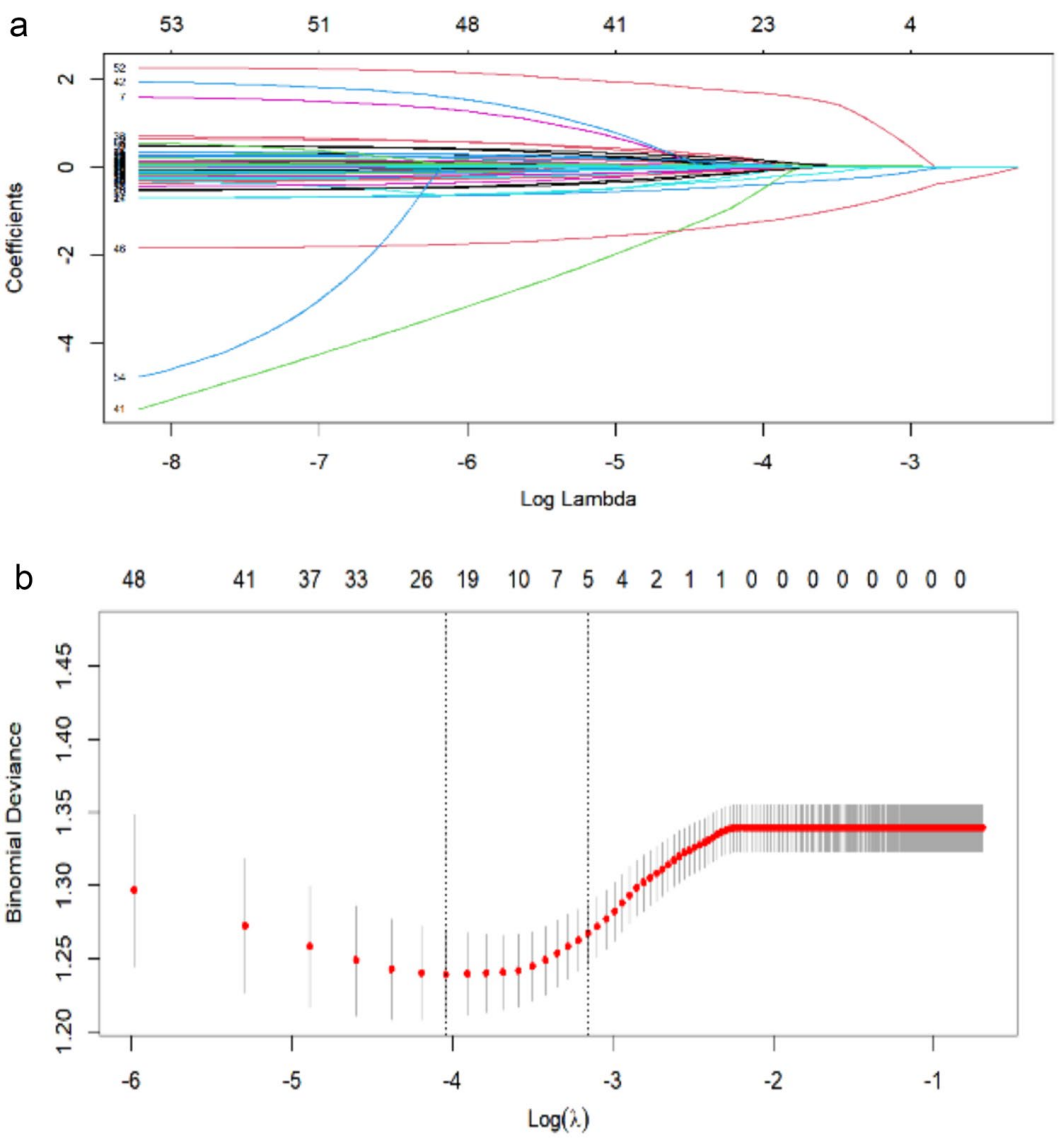
Variable selection by the LASSO binary logistic regression model

The study employed LASSO regression analysis to identify significant predictor variables from Table 1, followed by multivariate logistic regression to construct a prediction model. Out of the initial 53 variables, only 5 were included in the final prediction model. These variables, namely age, preoperative water abstinence time, MAP, SBP, and use of NE, were selected based on their non-zero coefficients in the LASSO regression model (Fig. 2). The prediction model was presented as a nomogram, providing a quantitative estimate of the probability of PACU hypotension risk in elderly patients undergoing sedated gastroenterology. The corresponding β-coefficients, OR with 95% CIs, and p-values are reported in Table 2.

**Table 2:** Logistic regression analysis of PACU hypotension risk predictors in elderly patients undergoing sedated gastrointestinal endoscopy

Intercept and variables	Estimate	z value	Prediction model		
			OR值	95%CI	*P* value
Intercept	−4.186	−4.219	0.015	0.002~0.105	<0.001
Age	0.033	2.409	1.033	1.006~1.062	**0.016**
Water abstinence time	0.073	4.442	1.076	1.042~1.112	**<0.001**
Intra-MAP	0.667	3.391	1.948	1.329~2.876	**<0.001**
SBP change	3.161	4.144	23.603	5.369~107.177	**<0.001**
Use of NE	−1.662	−8.019	0.19	0.125~0.283	**<0.001**

**Fig. 3:**
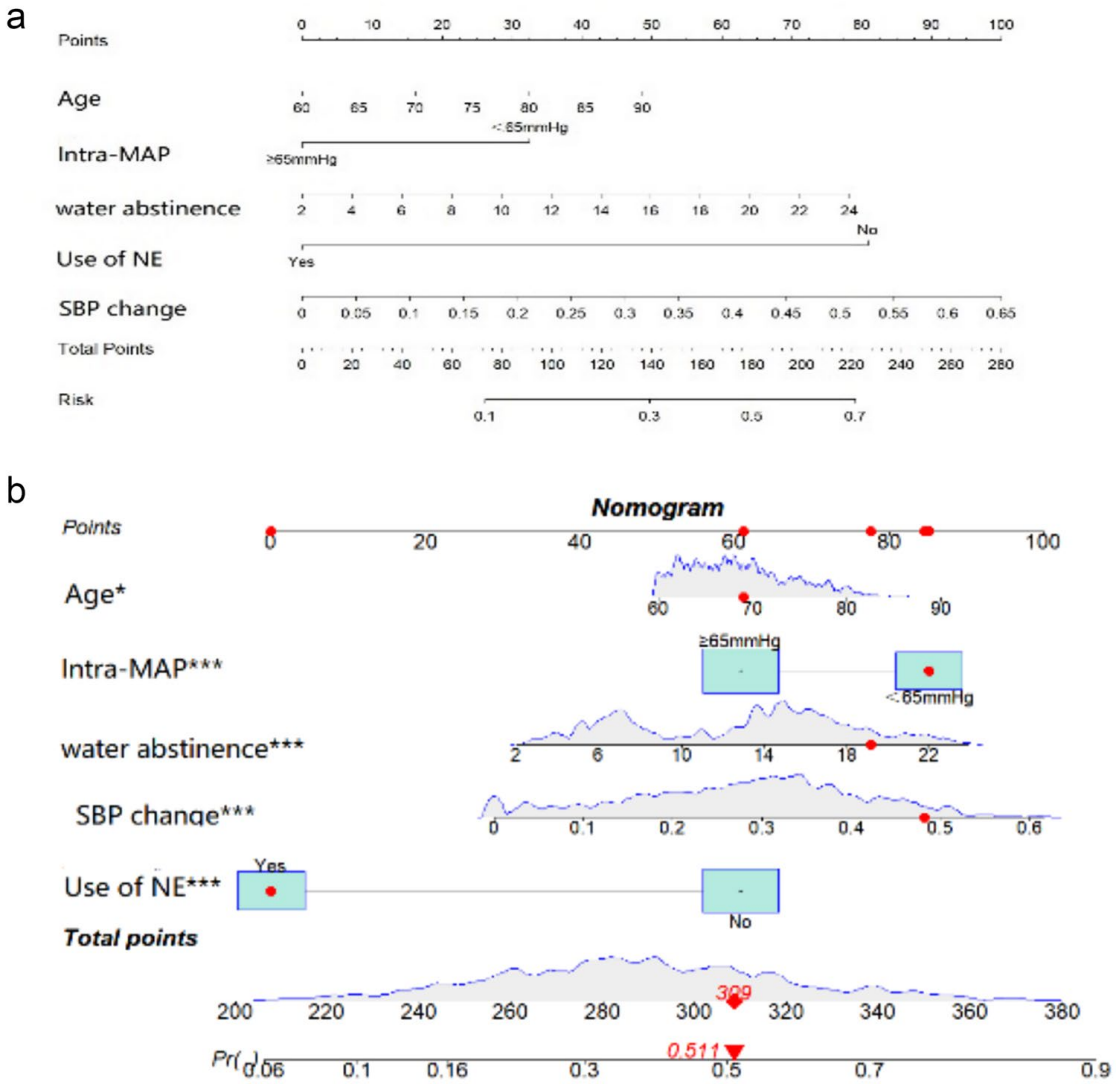
**a** Risk factors such as age, duration of preoperative water abstinence, intraoperative MAP <65 mmHg, decreased SBP, and the use of NE for the nomogram prediction model. **b** An example of a dynamic nomogram. The significance of the asterisks beside each variable in part (**b**) represents the importance of all risk factors

The results of the multivariable logistic regression analysis, delineated in Table 2, reveal statistically significant differences among all five predictors. Based on these independent predictive factors, a nomogram was constructed (Fig. 3a). This nomogram model was employed to estimate the probability of PACU hypotension in a geriatric patient aged 69 years, who underwent preoperative water abstinence for 19.52 h, experienced an intraoperative MAP <65 mmHg and a 46% decline in SBP, and was administered NE. The nomogram model yielded a probability estimate of 51.1% for PACU hypotension (Fig. 3b).

**Fig. 4:**
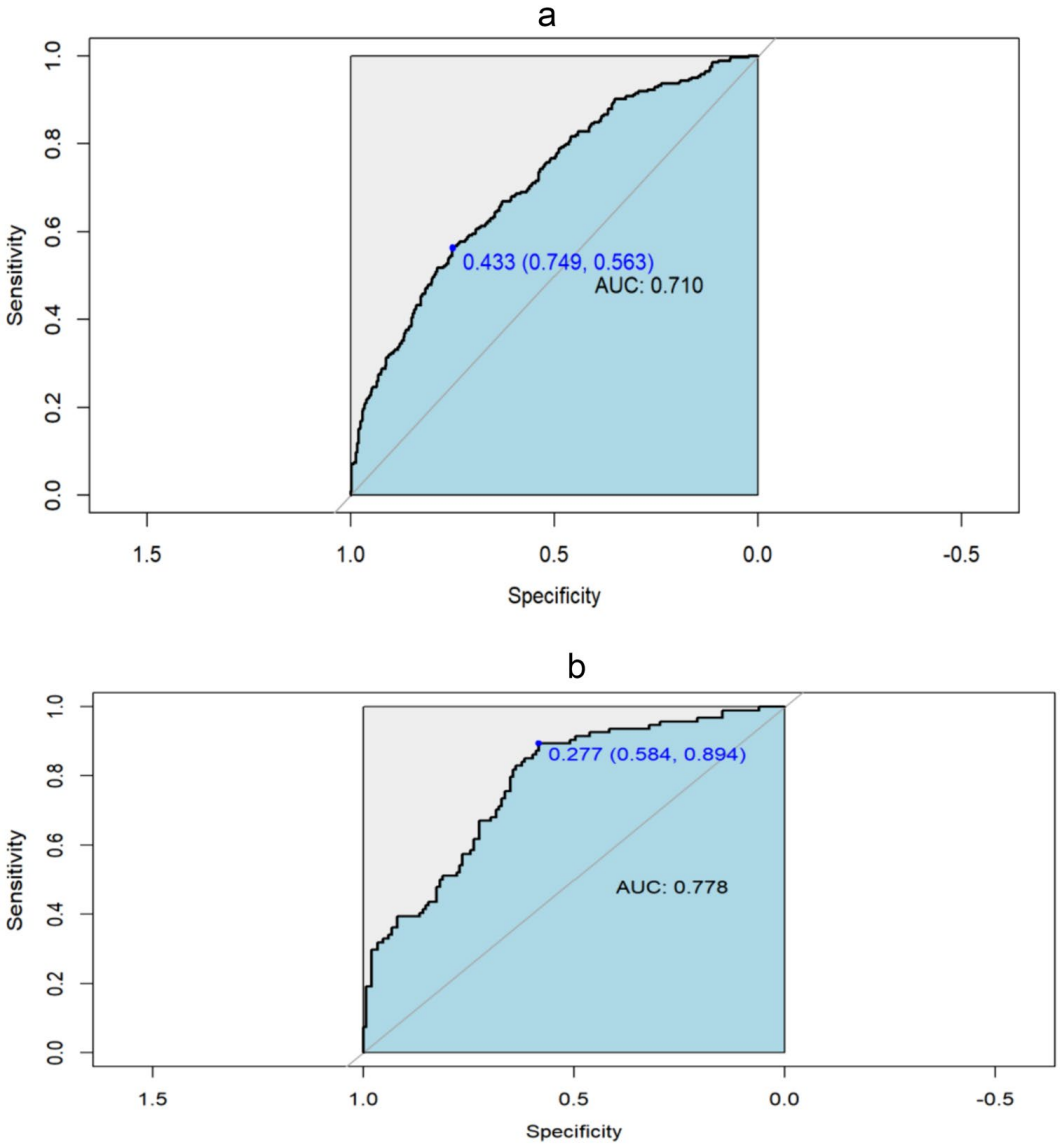
Receiver operating characteristic (ROC) curve validation of the PACU hypotension risk nomogram prediction

**Fig. 5:**
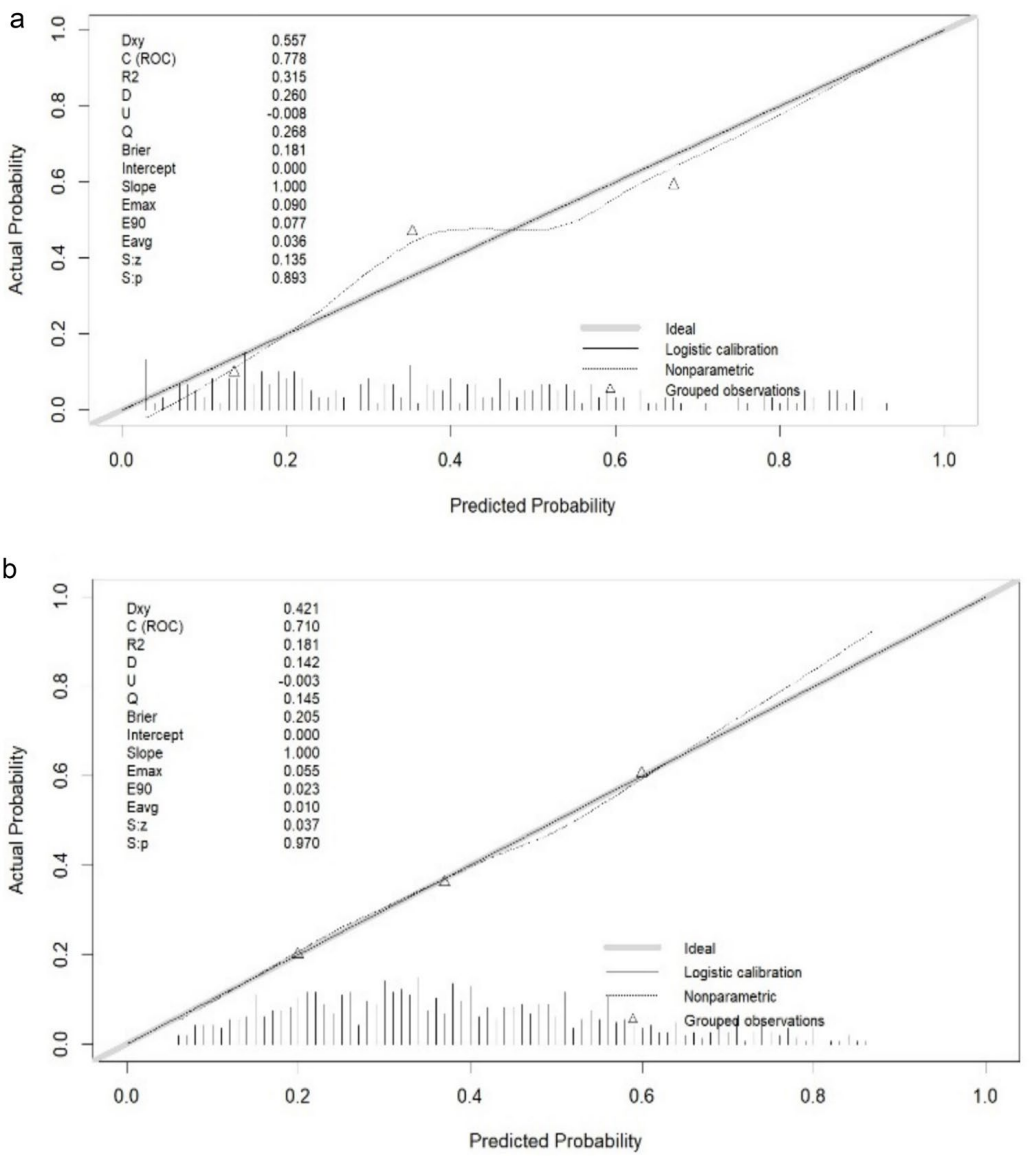
Calibration curves for the predictive PACU hypotension risk nomogram

**Fig. 6:**
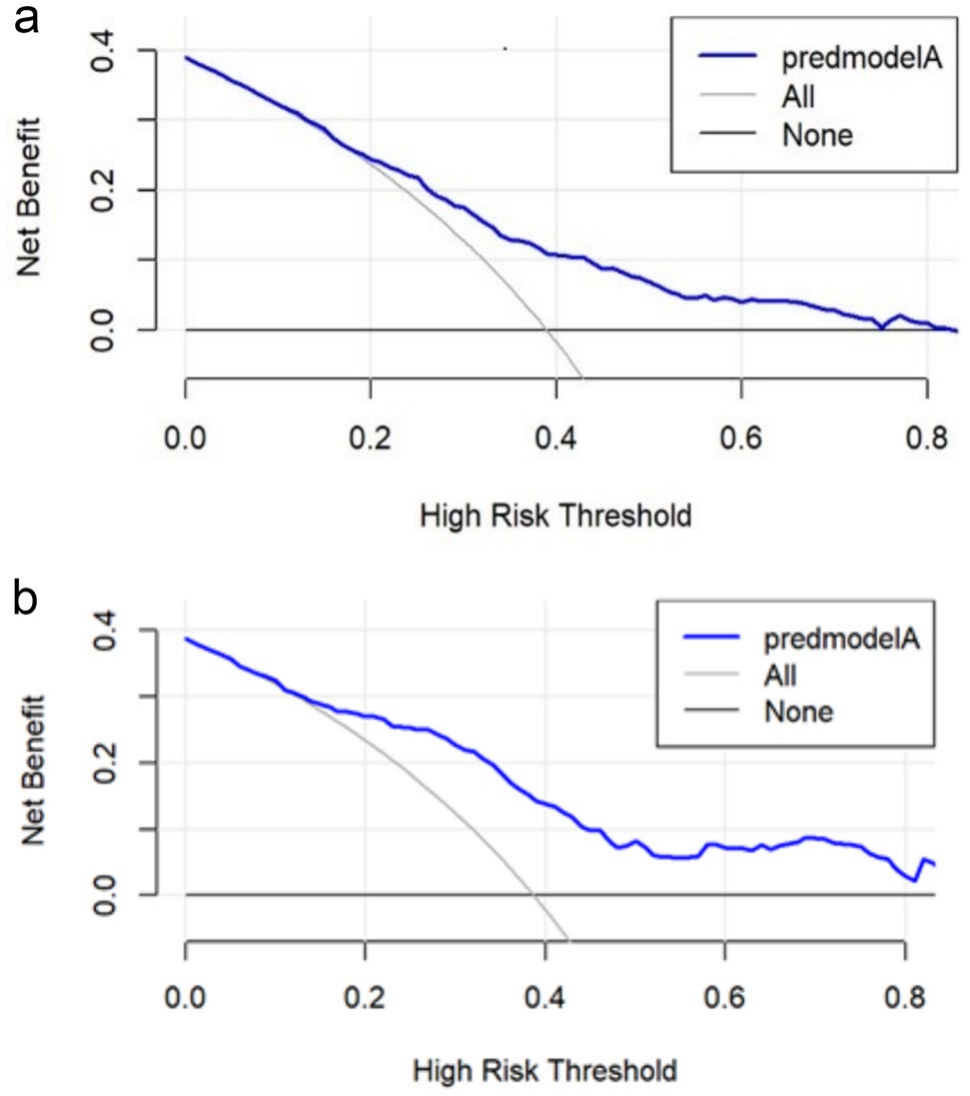
Decision curve analysis for the PACU hypotension risk nomogram

The discriminative ability of our model was assessed using ROC curves, which are commonly employed to evaluate the predictive capacity of models. The ROC analysis of our prediction model produced an AUC of 0.710 in the training set and 0.778 in the validation set, indicating moderately good performance (Fig. 4). Subsequently, a calibration plot and the Hosmer–Lemeshow test were used to refine the prediction model. The calibration curves revealed that the prediction model performed reasonably well in the validation set. Moreover, the Hosmer–Lemeshow test demonstrated high consistency between the predicted and actual probabilities, with *P* values of 0.893 and 0.970 in the training and validation sets, respectively (Fig. 5). Finally, the DCA indicated threshold probabilities of 18–90% and 20–82% in the training and validation sets, respectively, for the prediction model (Fig. 6).

A coefficient profile plot was constructed against the log (lambda) sequence. (a) 10 variables with non-zero coefficients were selected by deriving the optimal lambda.

The y-axis indicates the true positive rate of risk prediction, and the x-axis indicates the false positive rate of risk prediction. The thick black lines indicate the performance of the nomogram in the training set (a) and the validation set (b).

The y-axis represents the actual diagnosed cases of PACU hypotension, whereas the x-axis represents the predicted risk of PACU hypotension. The diagonal dotted line represents perfect prediction by an ideal model. The solid line represents the performance of the training set (a) and validation set (b), with the results indicating that a closer fit to the diagonal dotted line represents a better prediction.

The y-axis measures the net benefit. The thick solid line represents the assumption that no patients have PACU hypotension, whereas the thin solid line represents the assumption that all patients have PACU hypotension. The dotted line represents the risk nomogram results from the (a) training set and (b) validation set.

## Discussion

Using multivariate logistic regression analysis, a predictive model was constructed to determine the likelihood of postoperative hypotension in patients undergoing sedated gastroenteroscopy. The model demonstrated robust discrimination and calibration. Our findings indicate that several variables, including age, duration of preoperative water abstinence, intraoperative MAP, changes in SBP, and the use of NE, were identified as independent predictors of PACU hypotension based on the developed prediction model.

Numerous studies have investigated the factors associated with intraoperative and postoperative hypotension in general anesthesia, given its high incidence and close relation to postoperative recovery and adverse outcomes. Conversely, sedation anesthesia used during sedated gastroenteroscopy outside the operating room is characterized by a fast turnaround time, short treatment duration, and a disconnect between the medical and nursing staff and the available equipment. This mismatch, coupled with the reduced intensity of routine monitoring in the PACU, can result in delayed or missed diagnoses of perioperative hypotension [26–29]. Hypotension is now recognized as a common sedation-related adverse event during sedated gastroenteroscopy, particularly in elderly patients receiving propofol. Therefore, we randomly assigned our study population to two groups in a 3:1 ratio for external validation. Variables were initially screened using LASSO regression analysis, followed by traditional logistic regression analysis. Finally, ROC curves, calibration plots, and DCA were used to evaluate the model.

### Correlation between intraoperative blood pressure and PACU hypotension

Perioperative hypotension is commonly observed during outpatient, sedated gastrointestinal endoscopy sessions. A meta-analysis by Sneyd et al. [31] found that hypotension was prevalent during propofol sedation for colonoscopy, with longer sedation periods and higher doses of propofol associated with more sustained and profound hypotension. In the present study, postoperative hypotension was equally common and may have persisted for an extended period. Notably, many hypotensive events were not detected by standard routine monitoring systems. Given the association between postoperative hypotension and organ dysfunction and the ease with which hypotension can be overlooked in the outpatient PACU, further investigation into this critical period is warranted.

The definition of hypotension remains controversial; however, it is widely recognized that perioperative hypotension is associated with adverse postoperative outcomes, such as myocardial infarction, acute kidney injury, and stroke [26, 32–36]. While patients undergoing surgery receive continuous hemodynamic monitoring with ample opportunities for blood pressure management, most studies on intraoperative hypotension and its adverse outcomes do not address early postoperative hypotension [26–29]. Notably, intraoperative hypotension is strongly linked to postoperative hypotension, and an increase in the depth and/or duration of intraoperative hypotension can result in increased hypoxia-induced cellular injury. Such cellular injury can impair the systemic regulation of blood flow, potentially leading to prolonged and treatment-resistant intraoperative hypotension and increasing the likelihood of postoperative hypotension.

The results of this study suggest that an intraoperative decline in SBP and MAP <65 mmHg are independent risk factors for postoperative hypotension in the PACU. A Perioperative Quality Initiative consensus statement suggests that even brief periods of SBP and MAP below 60–70 mmHg in noncardiac surgery can be harmful [37]. Previous research has found [37] that both MAP and SBP are equally associated with cardiac and renal injury, with short exposures to MAP <55 mmHg or a brief reduction in SBP by 41–50 mmHg from the baseline being linked to myocardial injury and increased odds of myocardial infarction. Long-term exposure to MAP <80 mmHg or shorter exposure below 70 mmHg has been associated with a slightly elevated risk of end-organ damage [36]. Although our study did not analyze the direct correlation between postoperative hypotension and adverse outcomes, it is important to consider the potential impact of transient postoperative PACU hypotension on patients with postoperative myocardial, renal, or other injuries, in line with the theory of hypotension-induced ischemia–reperfusion injury [38].

### Correlation of age and PACU hypotension

Compared to their younger counterparts, older individuals are more susceptible to hypotension following anesthesia induction, particularly with the use of propofol [39]. This increased susceptibility suggests a decline in vascular self-regulation with advancing age. Furthermore, due to age-related degeneration of organ function, vital organs in older patients become less tolerant of hypotension, rendering them more vulnerable [40]. Consequently, hypotension-related complications, both direct and indirect, are frequently observed in the perioperative period, with postoperative mortality rates being 4.4 times higher in elderly patients than in young and middle-aged patients [41]. Managing perioperative anesthesia poses significant challenges owing to numerous risk factors, and maintaining blood pressure stability is a central goal of hemodynamic management [42]. Extensive retrospective observational studies have generated a substantial body of evidence, leading to clinical recommendations to avoid perioperative hypotension, especially in elderly patients [19, 36]. This recommendation stems from the vulnerability of elderly individuals to hypoperfusion and ischemia, as well as the physiological complexity of hemodynamic regulation. The Society of Anesthesiologists’ Best Practice Guidelines for Perioperative Care of the Elderly advises against intraoperative hypotension in patients aged ≥65 years, defining it as a 20% reduction in SBP [43].

### Correlation between preoperative water abstinence time and PACU hypotension

Preoperative water abstinence is an essential preventive measure to reduce the risk of aspiration during anesthesia, and guidelines recommend a 2–4-h water abstinence period for adult patients [44]. However, adherence to these guidelines can be challenging due to various factors that impede patients from following the recommended fasting period [45]. For instance, patients who undergo sedated gastroscopies are scheduled for morning procedures, leading to overnight fasting from the previous evening. Moreover, owing to unforeseen circumstances or a large patient volume, some patients may not be able to adhere to the recommended fasting period, leading to an extended period of water abstinence beyond 4 h. Notably, preoperative fasting and water abstinence may result in fluid loss, including non-obvious fluid loss, which is directly proportional to the duration of the fast [46]. Additionally, elderly patients are particularly susceptible to circulatory volume deficits and abnormalities in glucose metabolism, electrolytes, and the acid–base balance resulting from water abstinence [42]. As cardiovascular function declines with age, blood volume becomes critical in maintaining hemodynamic stability, and changes in blood volume can significantly affect blood pressure. In this study, the mean duration of preoperative water abstinence was observed to be 15.9 (range, 7.6–16.5) hours, which is consistent with previous studies [39, 47]. Notably, Morley et al. [39] observed little effect of the duration of preoperative water abstinence on MAP after propofol induction and concluded that dehydration resulting from prolonged water abstinence and extended fasting had a lesser effect on patient blood volume. The results of our study are different, probably because it included only healthy adults aged 18–64 years and did not focus on older adults. Children, another population susceptible to circulatory changes, have been studied by Simpao et al. [48] and Dennhardt et al. [49], who demonstrated that prolonged water abstinence has an adverse impact on hemodynamic stability in children after anesthesia. This suggests that prolonged water abstinence affects different populations to varying degrees. In elderly patients, prolonged water abstinence led to an increased incidence of postoperative hypotension, earlier onset of hypotension, and increased use of NE, as demonstrated in this study. Therefore, the importance of shortening water abstinence time should not be underestimated. Recent research has shown a growing consensus that preoperative consumption of clear fluids or carbohydrate beverages can enhance early postoperative recovery of gastrointestinal function and better maintain hemodynamic stability during the induction of anesthesia in patients undergoing elective gastrointestinal surgery, compared to intravenous rehydration [50].

### Correlation of intraoperative vasoactive drug use and PACU hypotension

This study demonstrates that the administration of vasoconstrictors during surgery can significantly reduce the incidence of postoperative hypotension in the PACU. In our gastroscopy clinic, routine intraoperative rehydration is uncommon, except in cases of patients with significant volume deficits. In such cases, vasoconstrictors are crucial for restoring vascular tone and maintaining MAP after replenishing average volume. Previous research has indicated that maintaining blood pressure within 10% of reference values can prevent postoperative organ dysfunction, compared to strategies that focus solely on treating SBP values below 80 mm Hg or less than 40% of the reference value [33]. However, owing to the short duration of gastroscopy exams and the reduced cardiovascular regulation in elderly patients, a single administration of boosting agents can result in transient blood pressure elevation and increased likelihood of PACU hypotension. Additionally, several factors, including variable definitions of hypotension, complicate the determination of optimal treatment for individual patients. McDonagh et al. [51] indicates that healthcare professionals have an inconsistent understanding of hypotension. For instance, some healthcare professionals consider hypotension as a fall in blood pressure below typical levels [52], while others consider a post-anesthesia drop in blood pressure relative to baseline, especially in patients with hypertension [53]. Failing to administer vasopressors even when blood pressure falls below 20% of the baseline can increase the risk of postoperative PACU hypotension, which can have serious patient outcomes. Prior studies have demonstrated that aggressive blood pressure control reduces organ damage and suggest a causal relationship between hypotension and at least 25% of significant complications [33].

The results of our study indicate that the utilization of model risk scores significantly enhances the performance of clinicians during the perioperative period, presenting a novel approach that provides a valuable tool for preventing PACU hypotension. This approach involves optimizing end-of-surgery care and preparing PACU staff to handle patients at risk of deterioration. Specifically, the incorporation of technology-based algorithms for informed decision-making can facilitate the development of proactive strategies, such as the establishment of protocols for more frequent postoperative vital sign monitoring in select patients, proactive continuation of intraoperative fluids or vasopressors in the PACU, optimization of PACU care tasks to address possible patient needs, and enhanced handoff discussions between operating room and PACU teams. These discussions should encompass the creation of hypotension treatment plans and the triggering of care escalation when necessary.

## Limitations

The current study has several limitations that should be acknowledged. Firstly, it employed a single-dosing regimen of propofol and remifentanil, which have been shown to cause hemodynamic fluctuations. This selection of induction drugs may have contributed to the occurrence of intraoperative and postoperative hypotension, potentially leading to false positive results. Secondly, the assessment of preoperative medical history was subjective, and the lack of objective tests and examination indicators may have compromised the accuracy of the results. Thirdly, the study did not include data on the long-term follow-up for patients after surgery, and it was conducted at a single center, limiting the generalizability of the findings. Future research should focus on examining the relationship between PACU hypotension, postoperative adverse events, and quality of life in a larger sample and across multiple centers.

## Conclusion

This analysis identified five significant indicators through the nomogram, namely age, duration of preoperative water abstinence, intraoperative MAP <65 mmHg, decreased SBP, and the use of NE. These indicators play a crucial role in identifying the risk of PACU hypotension in elderly patients undergoing sedated gastrointestinal endoscopy. The nomogram model, constructed based on these indicators, demonstrates a high degree of predictive power and clinical applicability for assessing the risk of PACU hypotension. This tool will enable clinicians to more effectively screen and evaluate the risk of PACU hypotension in elderly patients undergoing sedated gastrointestinal endoscopy. Furthermore, it will facilitate the implementation of targeted intervention measures for high-risk individuals, thus contributing to the prevention and treatment of PACU hypotension.

### Supplementary Information

Below is the link to the electronic supplementary material.

Supplementary file1 (DOCX 14 KB)

## Declarations

### Availability of data and materials

The datasets generated and analyzed during the current study are not publicly available due privacy issues, but are available from the corresponding author on reasonable request.
