# Supplementary material for: Development and validation of a predictive model for PACU hypotension in elderly patients undergoing sedated gastrointestinal endoscopy
Source: Aging Clin Exp Res. 2024 Jul 18;36(1):149. doi: 10.1007/s40520-024-02807-6 (PMC11258065; doi:10.1007/s40520-024-02807-6)
Supplement: Supplementary file 1 — Supplementary file1 (DOCX 14 KB) [file 40520_2024_2807_MOESM1_ESM.docx]

**Supplementary Table Incremental AUC Values for Each Parameter Added to the Model**

| **Model Parameters** | **AUC (Training Set)** | **AUC (Validation Set)** |
| --- | --- | --- |
| Age | 0.62 | 0.65 |
| Age + Preoperative water abstinence time | 0.65 | 0.68 |
| Age + Preoperative water abstinence time + MAP<65 mmHg | 0.68 | 0.72 |
| Age + Preoperative water abstinence time + MAP<65 mmHg + Decreased SBP | 0.7 | 0.75 |
| Age + Preoperative water abstinence time + MAP<65 mmHg + Decreased SBP + Use of NE | 0.71 | 0.78 |
